# Transcription factors FabR and FadR regulate both aerobic and anaerobic pathways for unsaturated fatty acid biosynthesis in *Shewanella oneidensis*

**DOI:** 10.3389/fmicb.2014.00736

**Published:** 2014-12-22

**Authors:** Qixia Luo, Miaomiao Shi, Yedan Ren, Haichun Gao

**Affiliations:** Institute of Microbiology and College of Life Sciences, Zhejiang UniversityHangzhou, China

**Keywords:** UFAs, biosynthesis, regulation, *Shewanella*, FadR

## Abstract

As genes for type II fatty acid synthesis are essential to the growth of *Escherichia coli*, its sole (anaerobic) pathway has significant potential as a target for novel antibacterial drug, and has been extensively studied. Despite this, we still know surprisingly little about fatty acid synthesis in bacteria because this anaerobic pathway in fact is not widely distributed. In this study, we show a novel model of unsaturated fatty acid (UFA) synthesis in *Shewanella*, emerging human pathogens in addition to well-known metal reducers. We identify both anaerobic and aerobic UFA biosynthesis pathways in the representative species, *S. oneidensis*. Uniquely, the bacterium also contains two regulators FabR and FadR, whose counterparts in other bacteria control the anaerobic pathway. However, we show that in *S. oneidensis* these two regulators are involved in regulation of both pathways, in either direct or indirect manner. Overall, our results indicate that the UFA biosynthesis and its regulation are far more complex than previously expected, and *S. oneidensis* serves as a good research model for further work.

## Introduction

Bacterial membrane, an essential structure involved in almost every aspect of bacterial growth and metabolism, generally lacks sterols and contains mainly phospholipids such as phosphatidylethanolamine (PtdEtn) and phosphatidylglycerol (PtdGro) (Zhang and Rock, 2008). To support normal structure and functions, membranes are required to maintain the appropriate fluidity, which is determined largely by the composition of fatty acids attached to membrane phospholipids, including straight-chain unsaturated fatty acids (UFAs), saturated fatty acids (SFAs), and branched-chain fatty acids (BCFAs) (Campbell and Cronan, 2001; Mendoza, 2014). As these fatty acids are required for bacterial survival, their biosynthesis pathway has been an important target for the development of novel antimicrobials in recent years, even though microbes are capable of incorporating environmental fatty acids into phospholipids (Campbell and Cronan, 2001; Parsons and Rock, 2011).

In bacteria, two pathways have been identified for biosynthesis of UFAs, distinct from each other in whether oxygen is involved (Altabe et al., 2013). The anaerobic pathway comprises, as best illustrated in *Escherichia coli*, a group of highly conserved proteins known as the type II fatty acid synthase (FAS II) system (Campbell and Cronan, 2001). During biosynthesis, the double bond is introduced at the C10 level by FabA, which catalyzes the dehydration and isomerization reactions to produce *cis*-3-decenoyl-ACP from standard biosynthetic intermediate, the *trans*-2-decenoyl thioester of acyl carrier protein (ACP) (Cronan et al., 1969). FabB, a β-ketoacyl-ACP synthase, is required to elongate the *cis*-3-decenoyl-ACP and its activity is the primary factor in determining cellular UFA content (Cronan, 2006). However, FabA and FabB in fact are not widely distributed in anaerobic bacteria that produce UFAs; as a consequence, a similar pathway with functional replacements of *E. coli* FabA and FabB is evolved (Marrakchi et al., 2002; Wang and Cronan, 2004). The aerobic pathway employs iron-containing transmembrane proteins to desaturate the fully elongated acyl chains, such as Des in *Bacillus subtilis* (Aguilar et al., 1998). While bacteria in which the subject has been investigated so far are generally equipped with either pathway for UFA biosynthesis, *Pseudomonas aeruginosa* has both, with two independent desaturases DesA and DesB (Zhu et al., 2006).

Given its critical role in maintaining membrane integrity and proper fluidity, it is conceivable that biosynthesis of fatty acids is tightly regulated in response to changes in environment. In *E. coli*, the most prominent control resides at the transcriptional level, which depends on FadR (fatty acid degradation regulator) and FabR (fatty acid biosynthesis regulator) (Fujita et al., 2007; Zhu et al., 2009). FadR, a regulator of the GntR family, principally functions as a global regulator of both UFA synthesis and the β-oxidative utilization of fatty acids as a carbon source and is antagonized by long-chain acyl-CoAs (Henry and Cronan, 1992; Cronan and Subrahmanyam, 1998). Its regulation on UFAs is realized by activating the *fabA* and *fabB* genes and by repressing the β-oxidation operons. FabR, discovered much later (McCue et al., 2001), is a regulator of the TetR family, which acts as a repressor for the *fabA* and *fabB* genes in the presence of UFAs (Zhu et al., 2009; Feng and Cronan, 2011). In the case of aerobic desaturases, information about their regulation is limited. In *B. subtilis*, a two-component system, DesK-DesR, mediates expression of the *des* gene in response to a decrease in environmental temperature (Cybulski et al., 2010). In *P. aeruginosa*, the *desB* gene is regulated by an *E. coli* FabR homolog DesT, which senses the physical properties of the cellular acyl-CoA pool, while regulation of the *desA* gene remains unknown (Zhang et al., 2007; Miller et al., 2010). Surprisingly, an *E. coli* FadR appears to be missing in *P. aeruginosa* given that it contains up to 27 GntR homologs (Choi and Schweizer, 2005).

*Shewanella* species are widely distributed in environments, and are well known for their versatile respiration capabilities, which are exploited for bioremediation of toxic elements and serving as microbial fuel cells (Fredrickson et al., 2008). In contrast to this beneficial role, Shewanellae are increasingly being implicated as human pathogens in persons exposed through occupational or recreational activities to marine niches where these species thrive (Janda and Abbott, 2014). Particularly, some *Shewanella* have been identified as gut pathogen, causing food poisoning by presumably producing tetrodotoxin, a potent neurotoxin that selectively blocks voltage-sensitive Na^+^ ion channels (Auawithoothij and Noomhorm, 2012; Moczydlowski, 2013; Wang et al., 2013). As the membrane composition of *Shewanella* is, at least in part, accountable for their widely distribution and unique physiological characteristics, in this study we took on to investigate the UFA biosynthesis and regulation in the intensively studied representative, *S. oneidensis*. We show that *S. oneidensis* possesses a FabA-based anaerobic UFA synthesis pathway and a single desaturase DesA for aerobic UFA biosynthesis, as well as two well-established regulators, FabR and FadR. Our results describe that the anaerobic pathway plays a predominant role in UFA synthesis and the aerobic pathway becomes active in the absence of FabA. We further show that FadR directly activates the anaerobic pathway, while FabR acts as a repressor for both pathways. In short, for the first time we provide insights into the regulation of UFA homeostasis in a bacterium equipped not only with both anaerobic and anaerobic pathways but also with FadR and FabR regulators.

## Methods and materials

### Bacterial strains, plasmids, and culture conditions

Bacterial strains and plasmids used in this study are listed in Table 1 and sequences of primers used are given in Table S1. All chemicals were acquired from Sigma Co. (Shanghai, China) unless specifically noted. For genetic manipulation, *E. coli* and *S. oneidensis* strains under aerobic conditions were grown in Luria-Bertani (LB) medium at 37 and 30°C, respectively. When needed, the growth medium was supplemented with chemicals at the following concentrations: 2,6-diaminopimelic acid (DAP), 0.3 mM; ampicillin, 50 μg/ml; kanamycin, 50 μg/ml; and gentamycin, 15 μg/ml.

**Table 1:** **Strains and plasmids used in this study**.

**Strain or plasmid**	**Relevant characteristics**	**Sources or references**
***E. COLI* STRAINS**		
DH5α	Host strain for plasmids	Lab stock
WM3064	Donor strain for conjugation; Δ*dapA*	W. Metcalf, UIUC
XL1-Blue MRF'Kan	Recipient strain for one-hybrid system	Stratagene
***S. ONEIDENSIS* STRAINS**		
MR-1	Wild-type	ATCC 700550
HG0197	Δ*desA* derived from MR-1 As MR-1 plus Δ*katG-1*	This study
HG0198	Δ*fabR* derived from MR-1	This study
HG1856	Δ*fabA* derived from MR-1	This study
HG2885	Δ*fadR* derived from MR-1	This study
HG0197-1856	Δ*fabA*Δ*desA* derived from MR-1	This study
**PLASMIDS**		
pHGM01	Suicide vector for mutant construction	Jin et al., 2013
pHG101	Promoterless vector for complementation	Wu et al., 2011
pHG102	pHG101 containing P*_ArcA_*	Wu et al., 2011
pHGEI01	Integrative *lacZ* reporter vector	Fu et al., 2014a
pBBR-Cre	Helper plasmid for antibiotic marker removal	Fu et al., 2013
pBXcmT	B1H bait vector	Guo et al., 2009
pTRG	B1H target vector	Stratagene
pTP247	His_6_-tag expression Vector, Ap^R^	Gao et al., 2008a

For physiological characterization, both LB and M1-defined medium containing 0.02% (w/v) of vitamin free Casamino Acids and 15 mM lactate as the electron donor were used in this study and consistent results were obtained (Gao et al., 2008a). Anaerobic growth was supported by 20 mM fumarate as the electron acceptor. Fresh medium was inoculated with overnight cultures grown from a single colony by 1:100 dilution, and growth under aerobic and anaerobic conditions was determined by recording the optical density of cultures at 600 nm (OD_600_). For cultures with fatty acid additions, which interfere with OD readings, growth was monitored by photographing colonies on plates. Mid-log-phase (~0.4 of OD_600_, unless mentioned otherwise) cells were properly diluted, plated on solid agar plates containing a paper disk of 6 mm in diameter as the size reference, and incubated at 30°C.

### In-frame deletion mutagenesis and complementation

In frame deletion strains were constructed according to the *att*-based Fusion PCR method described previously (Jin et al., 2013). In brief, two fragments flanking the gene of interest were amplified with primers containing *attB* and the gene specific sequence, and then joined by a second round of PCR. The fusion fragment was introduced into pHGM01 by site-specific recombination using the BP Clonase (Invitrogen) and maintained in *E. coli* WM3064. The resulting mutagenesis vector was then transferred from *E. coli* into *S. oneidensis* by conjugation. Integration of the mutagenesis construct into the chromosome was selected by gentamycin resistance and confirmed by PCR. Verified trans-conjugants were grown in LB broth in the absence of NaCl and plated on LB supplemented with 10% sucrose. Gentamycin-sensitive and sucrose-resistant colonies were screened by PCR for the intended deletion. The deleted mutants were then verified by sequencing.

Plasmids pHG101 and pHG102 were used in genetic complementation of mutants (Wu et al., 2011). For complementation of genes adjacent to their promoter, a fragment containing the gene of interest and its native promoter was generated by PCR and cloned into pHG101. For the rest genes, the gene of interest was amplified and inserted into MCS of pHG102 under the control of the *arcA* promoter, which is constitutively active (Gao et al., 2010a). After verified by sequencing, the vectors were introduced into the relevant mutants for phenotypic assays.

### Expression assays

Expression of genes of interest was assessed using an integrative *lacZ*-reporter system (Fu et al., 2014a). Based on the promoter prediction, fragments of ~300 bp covering the promoter sequences were cloned into the reporter vector pHGEI01 to generate transcriptional fusions. The resultant vectors were then verified by sequencing and then transferred into relevant strains by conjugation. To eliminate the antibiotic marker, helper plasmid pBBR-Cre was transferred into the strains carrying a correctly integrated construct (Fu et al., 2013). Mid-log phase cultures were harvested, properly aliquotted, and subjected to β-Galactosidase activity assay as described before (Fu et al., 2014a).

Expression of genes of interest was also assessed using quantitative reverse-transcription PCR (qRT-PCR). Cells of the mid-log phase were harvested by centrifugation and total RNA was isolated using RNeasy Mini Kit (QIAGEN) according to the manufacturer's instructions. The analysis was carried out with an ABI7300 96-well qRT-PCR system (Applied Biosystems) as described previously (Yuan et al., 2011).

### Fatty acid compositional analysis

To determine fatty acid composition, cultures of the mid-log phase grown in LB medium were collected by centrifugation, properly aliquotted, and subjected to total cellular lipid extraction as described before (Bligh and Dyer, 1959). The fatty acid methyl esters (FAMEs) were prepared by trans-esterification with 0.5 M sodium methoxide in methanol and identified using gas chromatograph-mass spectroscopy (GC-MS) (Focus GC-DSQ II) on a capillary column (30 mm by 0.25 mm in diameter) (Zhang et al., 2002). Helium at 1 ml/min was used as the carrier gas, and the column temperature was programmed to rise by 4°C/min from 140 to 170°C, and then 3.5°C/min from 170 to 240°C for 12.5 min.

### Bacterial one-hybrid (B1H) assay

B1H system was utilized to investigate DNA-protein interaction *in vivo* in *E. coli* cells as described previously (Guo et al., 2009; Jiang et al., 2014). Plasmids were constructed by cloning the “bait” promoter region DNA and “target” regulators into the pBXcmT and pTRG vectors, respectively. After verified by sequencing, the resultant plasmids were used to co-transform BacterioMatch II Validation Reporter Competent cells on M9 salt agar plates containing 25 mg/ml chloramphenicol and 12.5 mg/ml tetracycline with or without 3-amino-1,2,4-triazole (3-AT).

### Expression and purification of FadR_*SO*_

The cloning of *S. oneidensis fabR* and *fadR* for expression and purification has been described previously (Gao et al., 2008b). Soluble His-tagged FadR protein was expressed in *E. coli* BL21(DE3) induced with 0.3 mM isopropyl β-D-1-thiogalactopyranoside (IPTG) at 30°C for 4 h. Cells were collected by centrifugation, and resuspended in the lysis buffer (50 mM Tris/HCl, pH 7.5, 200 mM NaCl, 1 mM MgCl_2_, 10 mM β-mercaptoethanol, 1 mM PMSF, 5 mg/ml DNase I), and subjected to thorough sonication. The soluble protein was purified using a nickel-ion affinity column according to Ni-NTA purification system manual (Invitrogen).

### Electrophoretic mobility shift assay (EMSA)

Biotin-labeled DNA probes were prepared by PCR from *S. oneidensis* genomic DNA with biotin-labeled primers and EMSA was carried out as described before (Gao et al., 2008a). Briefly, the binding reaction was performed with ~2–5 nM labeled probes and various amount of protein in 12 μl binding buffer containing 100 mM Tris/HCl (pH 7.4), 20 mM KCl, 10 mM MgCl_2_, 2 mM DTT, 0.2 μg/μl poly(dI·dC), and 10% glycerol at 15°C for 60 min. Reaction mixtures were immediately resolved on 6% polyacrylamide native gels, blotted onto nylon membranes, and cross-linked with a UV crosslinker. The imaging procedure followed Thermo Scientific's chemiluminescent nucleic acid detection module and was visualized with the UVP Imaging System.

### Bioinformatics and statistical analyses

Promoter prediction for genes of interest was performed by using promoter prediction program Neural Network Promoter Prediction (Reese, 2001). For statistical analysis, values are presented as means ± SD (standard deviation). Student's-test was performed for pairwise comparisons of groups.

## Results

### *S. oneidensis* possesses both anaerobic and aerobic UFA biosynthesis pathways

**Figure 1:**
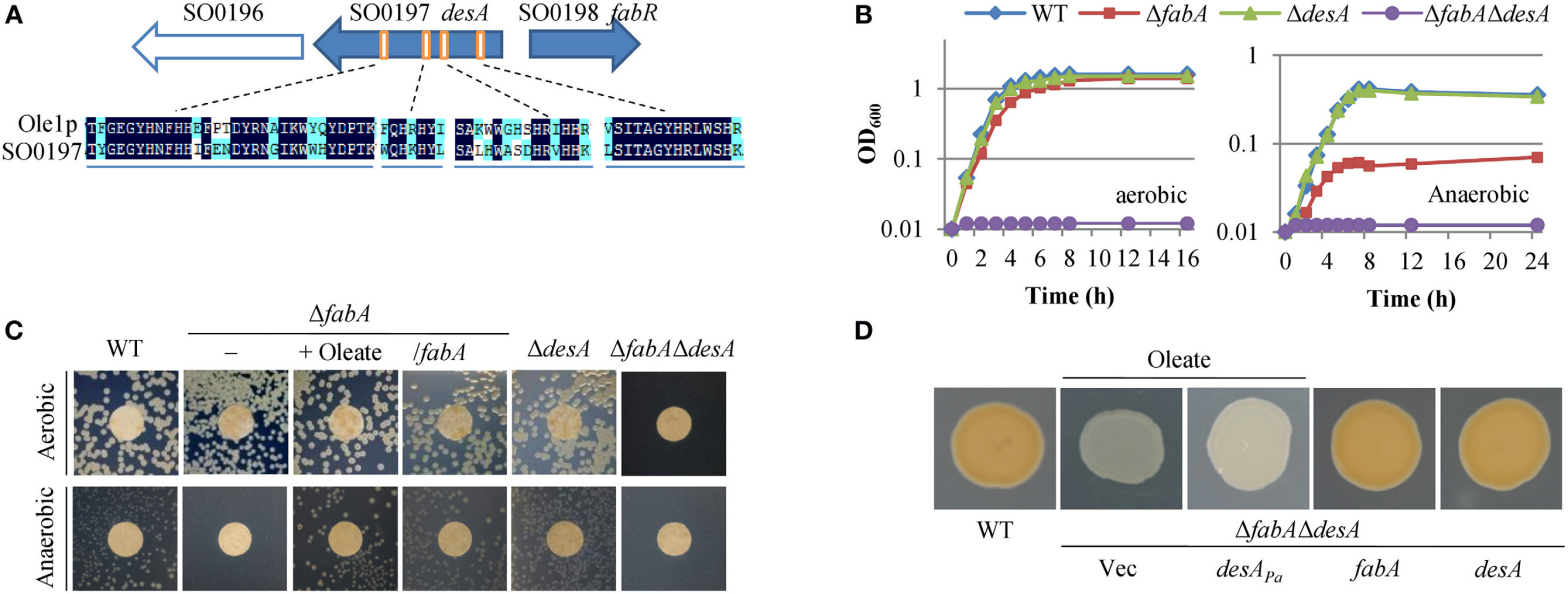
***S. oneidensis* possesses both anaerobic and aerobic UFA biosynthesis pathways**. **(A)** Genetic organization of *desA_So_* (SO0197) and comparison of the fatty acyl desaturase conserved histidine clusters, characteristic of desaturases. *S. cerevisiae* acyl-CoA desaturase (Ole1p) and DesA_So_ are aligned. Identical amino acids are highlighted in dark blue and similar amino acids are highlighted in light blue. **(B)** Growth of Δ*fabA_So_*, Δ*desA_So_*, and Δ*fabA_So_* Δ*desA_So_* strains in liquid media under aerobic and anaerobic conditions. Genetic complementation results are given in Figure S1. **(C)** Growth of Δ*fabA_So_*, Δ*desA_So_*, and Δ*fabA_So_*Δ*desA_So_* strains on solid media. Cultures of the mid-log phase for each strain were properly diluted, placed on LB plates, incubated for 24 and 48 h under aerobic and anaerobic conditions, respectively. Δ*fabA_So_* was complemented by chemically (supplement of oleate) or genetically (expression of *fabA_So_ in trans*). **(D)** Colony color phenotype of mutants defective in UFA synthesis. In order to support growth of Δ*fabA_So_*Δ*desA_So_*, oleate was supplemented when necessary. The double mutant was complemented by *desA_Pa_, fabA_So_*, or *desA_So_ in trans*, with empty vector (Vec) as control. Δ*fabA_So_* and Δ*desA_So_* strains were indistinguishable from WT (not shown). In **(B–D)** experiments were conducted independently at least three times and similar results were obtained **(C,D)** or standard deviations (less than 5% of the means) were omitted for clarity **(B)**.

According to the genome annotation, *S. oneidensis* has the *fabA_So_* (*SO1856*) (for differentiation, the same genes/proteins from multiple bacteria are labeled with abbreviated bacterial name in subscript) gene (Heidelberg et al., 2002), whose protein product shares up to 67% of sequence identity with FabA_Ec_ from *E. coli*. The gene is perfectly conserved in all sequenced *Shewanella* (data not shown), indicating that this group of bacteria own a typical anaerobic UFA synthesis pathway. However, genes encoding aerobic desaturases are not annotated. To assess whether *S. oneidensis* can also produce UFAs via an aerobic pathway, we screened the proteome with three well-studied desaturases essential to the process, Ole1p of *S. cerevisiae*, DesA of *P. aeruginosa* (DesA_Pa_), and Des of *B. subtilis* (Stukey et al., 1990; Aguilar et al., 1998; Zhu et al., 2006). BLASTp returned the same single putative homolog against Ole1p and DesA_Pa_, SO0197 (*E*-values 3e-64 and 2e-11, respectively). This protein shares sequence identity of 41% with the N-terminal desaturase domain of Ole1p, which also carries a C-terminal domain that has strong homology to cytochromes *b*_5_ (Mitchell and Martin, 1995). Like Ole1p, SO0197 bears characteristics of membrane-bound desaturases, three trans-membrane domains and four histidine-rich clusters (Figure 1A). These features suggest that *S. oneidensis* may utilize SO0197 as an aerobic fatty acyl desaturase.

To confirm that FabA_So_ and SO0197 function in the *S. oneidensis* UFA synthesis, in-frame deletion strains for their coding genes, individually and in combination, were constructed and assayed for growth under aerobic and anaerobic conditions. Both single knockout strains, Δ*fabA_So_* and Δ*SO0197*, were obtained on LB plates. The Δ*SO0197* strain was indistinguishable from the wild-type with respective to growth in liquid and solid media under aerobic and anaerobic conditions (Figures 1B,C). In contrast, the Δ*fabA_So_* strain not only grew at a reduced rate under aerobic conditions but also required a UFA supplement, oleate of 0.005% (the same concentration was used through this study unless otherwise noted), for anaerobic growth. These growth defects were corrected when a copy of *fabA_So_* was expressed *in trans* (Figure S1), confirming the essential role of FabA_So_ in anaerobic UFAs biosynthesis. However, we failed to remove *SO0197* in the absence of *fabA_So_* under routine conditions for mutagenesis. Given that the resolution step of the mutagenesis protocol theoretically gives the wild-type and mutant strains at 50:50 (Jin et al., 2013), the failure implicates a synthetical lethality under experimental conditions. We therefore reasoned that exogenous oleate may support growth of strains lacking both pathways. Indeed, when it was supplied, the Δ*fabA_So_*Δ*SO0197* strain was obtained. Its dependence on exogenous oleate was confirmed by the observation that the double mutant could not grow without the addition (Figures 1B,C). Neither palmitate (C16:0) nor decanoate (C10:0) was able to rescue the synthetical lethal phenotype, suggesting that there unlikely exists an additional desaturase in *S. oneidensis* that can replace the function of missing enzymes (data not shown). When complemented by any of the deleted genes *in trans*, the Δ*fabA_So_*Δ*SO0197* strain gained the ability to grow without exogenous oleate (Figure S1). These observations manifest that in the absence of FabA_So_ SO0197 is a desaturase capable of generating sufficient UFAs to support growth. Given that the amino acid sequence similarity of SO0197 and DesA_Pa_ is significantly higher than that of SO0197 and the other *P. aeruginosa* desaturase DesB_Pa_ (ClustalW2 score: 15.49 and 6.25, respectively), we named *SO0197* as *desA_So_*. Unexpectedly, as shown in Figure 1D the double mutant strain lost the orange color, a signature feature of *S. oneidensis* because of abundant *c*-type cytochromes (Gao et al., 2010b; Jin et al., 2013), and the wild-type color was restored when either gene was expressed *in trans*. Given that the microorganism produces more than 40 such proteins (Meyer et al., 2004; Gao et al., 2010b; Fu et al., 2014b), this finding suggests that UFAs are crucial for cytochrome *c* biosynthesis, which is currently under study.

It is worth mentioning that DesA_Pa_ was not able to rescue the synthetical lethal phenotype resulting from losing both pathways when its coding gene was expressed under the control of the *S. oneidensis arcA* promoter, which is constitutively active (Gao et al., 2010a; Dong et al., 2012; Zhang et al., 2013; Sun et al., 2014) (Figure 1D). Thus, although both DesA proteins act as desaturases, they are not functionally exchangeable, presumably due to low sequence similarity. Moreover, *S. oneidensis* differs from *P. aeruginosa* in that the latter has an additional desaturase, DesB_Pa_, which is proposed to selectively desaturates fatty acids from the environment (Zhu et al., 2006).

### *S. oneidensis* FadR and FabR are involved in regulation of both anaerobic and aerobic UFA synthesis pathways

In *E. coli*, regulation of the anaerobic UFA biosynthesis pathway is carried out by FadR_Ec_ and FabR_Ec_ (Fujita et al., 2007), whose counterparts in *S. oneidensis* are apparent, FadR_So_ (SO2885, *E*-value, 2e-82) and FabR_So_ (SO0198, *E*-value, 9e-60). This is in sharp contrast to *P. aeruginosa*, the only bacterium which has both anaerobic and aerobic UFA biosynthesis pathways and has been studied. A BLASTp search against the *P. aeruginosa* proteome using FadR_Ec_ returned many hits but none of them appeared to be a possible homolog (the smallest *E*-value, 2e-06). Moreover, the deletion of 25 of 27 GntR homologs did not significantly influence the bacterial response to exogenous fatty acids, suggesting that *P. aeruginosa* may not utilize a GntR family regulator for the role of FadR_Ec_ in *E. coli* (Choi and Schweizer, 2005). In *P. aeruginosa*, while little is known about how the *desA_Pa_* gene is regulated, the *desB_Pa_* gene is under direct control of DesT_Pa_, a homolog of FabR_Ec_ (Zhang et al., 2007). Consistently, a BLASTp search using DesT_Pa_ against *S. oneidensis* revealed a single putative homolog, FabR_So_ (*E*-value, 1e-22). The *desA_So_* and *fabR_So_* genes form a devergon (Figure 1A), a feature shared by the *desB_Pa_* and *desT_Pa_* genes, implicating a possibility that they may be physiologically associated.

**Figure 2:**
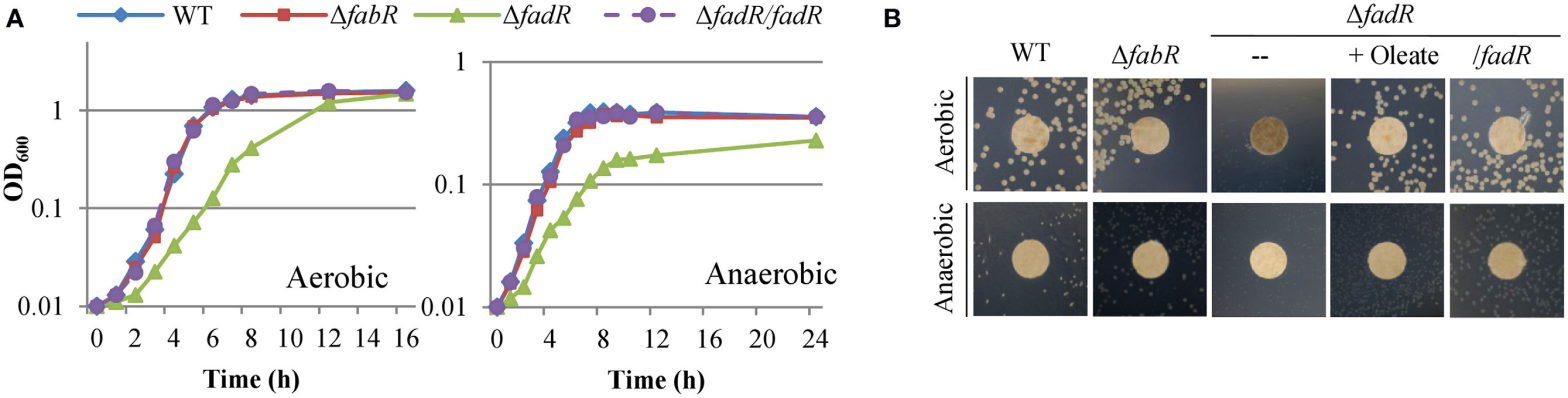
***S. oneidensis* possesses both FabR_So_ and FadR_So_ regulators**. **(A)** Growth of Δ*fabR_So_* and Δ*fadR_So_* strains in liquid media under aerobic and anaerobic conditions. **(B)** Growth of Δ*fabR_So_* and Δ*fadR_So_* strains on solid media. Complementation for Δ*fadR_So_* was carried out by chemically (supplement of oleate) or genetically (expression of *fadR_So_ in trans*). Experiments were conducted independently at least three times and similar results were obtained **(B)** or standard deviations (less than 5% of the means) were omitted for clarity **(A)**.

To confirm the involvement of FadR_So_ and FabR_So_ in UFA biosynthesis, mutants devoid of one of these genes were constructed. Under aerobic conditions, the Δ*fadR_So_* strain grew significantly slower than the wild-type; its generation time (~70 min) was nearly doubled (Figure 2A). Similar results were obtained from anaerobic cultures, indicating that FadR_So_ influences both anaerobic and aerobic UFA biosynthesis pathways. The observed defect in growth was corrected by expressing the *fadR_So_* gene *in trans* under both conditions (Figure 2A), validating that the phenotype was due to the intended mutation. Importantly, the defect resulting from the *fadR_So_* deletion was also fully eliminated by adding oleate to the medium (Figure 2B), offering direct evidence that the *fadR_So_* mutation affects the synthesis of UFAs. In contrast, the effect of deletion of the *fabR_So_* gene on growth under either aerobic or anaerobic conditions was insignificant. Moreover, the loss of either regulator did not significantly affect the colony color (data not shown). Overall, these data indicate that FadR_So_ has a more profound influence on UFA biosynthesis whereas FabR_So_ plays a dispensable role, at least in the context of mutant phenotypes.

### Loss of FabA or FadR significantly affects fatty acid composition

To gain an understanding of impacts of UFA biosynthesis pathways on fatty acids composition of *S. oneidensis*, the membranes of the wild-type, Δ*fabA_So_*, Δ*desA_So_*, Δ*fabR_So_*, and Δ*fadR_So_* strains were collected and assayed by GC-MS (Table 2). In the wild-type, SFAs, dominated by branched C15:0 (including both iso-C15:0 and antiiso-C15:0) and C16:0, accounted for ~67% of the total membrane lipids whereas the remaining consisted of three UFAs, with C16:1 as the major. Loss of DesA_So_ had little impact on the composition, consistent with the accessory role that it plays in UFA biosynthesis. Similar results were obtained from the Δ*fabR_So_* strain. Along with the observation that the loss of the *fabR_So_* gene does not elicit a distinguishable phenotype, these data indicate that the regulator is not a critical factor influencing the fatty acid composition of the *S. oneidensis* membrane. In contrast, removal of either *fabA_So_* or *fadR_So_* resulted in significantly altered fatty acid profiles. It was immediately evident that the percentages of iso-C15:0 and/or antiiso-C15:0 in both mutants increased substantially, especially in Δ*fadR_So_* (up to ~52%). Additionally, levels of C14:0 were elevated up to 3-fold although its absolute abundance (up to 10%) was low compared to branched C15:0. As a consequence, most of other fatty acids, such as C15:0, C16:1, C16:0, and C18:1, were present in reduced levels. The level of UFAs in the Δ*fabA* strain was ~77% relative to that in the wild-type, contrasting unaffected UFA biosynthesis in the absence of the *desA* gene. This observation further confirms that the anaerobic pathway dictates UFA biosynthesis. Moreover, the loss of FadR_So_ introduced a most drastic reduction in overall UFA levels, ~48% relative to the wild-type level. These data conclude that FadR_So_ is crucial to UFA biosynthesis and its impact probably goes beyond the anaerobic pathway.

**Table 2:** **Fatty acid composition of *S. oneidensis* strains**.

**Strain**	**C14:1**	**C14:0**	**i-C15:0**	**C15:0**	**C16:1**	**C16:0**	**C18:1**	**C18:0**
WT	1.98 ± 0.47	3.17 ± 0.78	22.60 ± 0.47	3.57 ± 0.31	27.06 ± 5.62	29.45 ± 7.64	4.09 ± 0.62	8.08 ± 2.65
Δ*fabA*	1.85 ± 0.94	7.55 ± 0.16	34.06 ± 9.54	5.05 ± 0.94	21.58 ± 3.75	22.11 ± 4.54	2.06 ± 0.16	5.74 ± 0.31
Δ*desA*	2.42 ± 1.22	3.93 ± 0.35	21.27 ± 4.00	3.46 ± 0.52	25.92 ± 3.83	31.55 ± 7.13	4.63 ± 2.09	6.82 ± 0.35
Δ*fabR*	3.01 ± 1.45	2.62 ± 0.81	21.68 ± 8.41	2.51 ± 0.65	30.55 ± 8.73	27.51 ± 7.44	6.11 ± 3.39	6.01 ± 2.10
Δ*fadR*	4.14 ± 0.29	9.77 ± 1.59	52.71 ± 13.7	0.71 ± 0.04	10.65 ± 1.30	15.31 ± 4.76	1.46 ± 0.43	5.25 ± 1.59
Δ*fabA*Δ*desA*^a^	2.55 ± 0.82	4.81 ± 0.96	19.69 ± 8.65	0.67 ± 0.14	14.43 ± 1.51	26.73 ± 2.61	25.83 ± 3.4	5.29 ± 0.92

a *0.005% oleate supplemented in culture*.

### The absence of the anaerobic pathway induces an enhanced transcription of the desA gene

**Figure 3:**
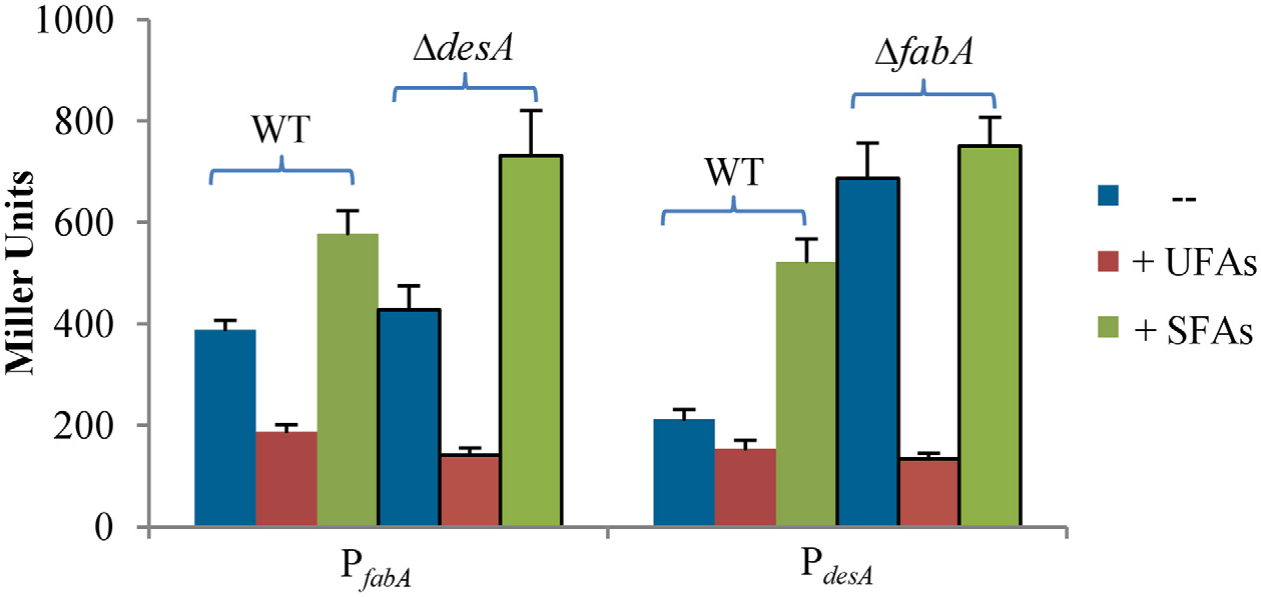
**Expression of *fabA_So_* and *desA_So_* under various conditions**. The *fabA_So_* and *desA_So_* promoters were placed in front of *E. coli lacZ* gene and integrated into the chromosome of the relevant strains. Their activity in a single copy in cells of mid-log phase was assayed by measuring β-galactosidase activity, presented in Miller unit. Effects of exogenous UFAs and SFAs were assessed. P*_fabA_* and P*_desA_* activities in Δ*desA_So_* and Δ*fabA_So_* strains were compared to that in the wild-type, respectively. Experiments were conducted independently at least three times and standard deviations were presented as error bars.

Results presented thus far indicate that the anaerobic pathway is critical to UFA biosynthesis in *S. oneidensis*. However, cells retain ability to produce a considerable amount of UFAs in its absence, implicating a possibility that the aerobic pathway enlarges its role when the anaerobic pathway is gone. To test this, a *lacZ* reporter system was employed to assess the activities of the *fabA_So_* and *desA_So_* promoters in the wild-type, Δ*fabA_So_*, and Δ*desA_So_* strains (Fu et al., 2014a). For both genes, the most confident promoters, predicted by using Neural Network Promoter Prediction (NNPP) (Reese, 2001), are within 100 bp upstream of their coding sequence. Proper reporter vectors were constructed by placing fragments of ~300 bp covering the predicted promoters in front of the full length *E. coli lacZ* gene and introduced into the relevant strains. After chromosome integration and the antibiotic marker removal, activities of these two promoters in a single copy were assayed (Figure 3). When grown under aerobic conditions, the activity of the *fabA_So_* promoter in the Δ*desA_So_* strain increased slightly, compared to that in the wild-type. In contrast, deletion of the *fabA_So_* gene caused an elevation of ~3.5-fold for the *desA_So_* promoter activity. Similar observations were obtained by using qRT-PCR (data not shown), indicating a complement role of the aerobic pathway for UFA production when the anaerobic pathway is absent.

We then evaluated influences of oleate (UFA) and palmitate (SFA) on these two UFA biosynthesis pathways. Activities of the *desA_So_* and *fabA_So_* promoters were assayed in cells grown with addition of either the fatty acid (Figure 3). In the wild-type background, exogenous oleate reduced *fabA_So_* promoter activity to approximately a half but showed a modest impact on expression of the *desA_So_* gene. Similar effects were observed from the *fabA_So_*, and *desA_So_* mutant strains. Notably, the induction of the *desA_So_* gene upon removal of FabA_So_ was no longer evident. On the contrary, the SFA addition induced both genes substantially, largely independent of FabA_So_ or DesA_So_. These results manifest that the supplement of UFAs suppresses expression of both UFA synthesis pathways whereas SFAs confer an opposite effect.

### Direct roles of FadR_SO_ and FabR_SO_ in regulation of UFA synthesis pathways

**Figure 4:**
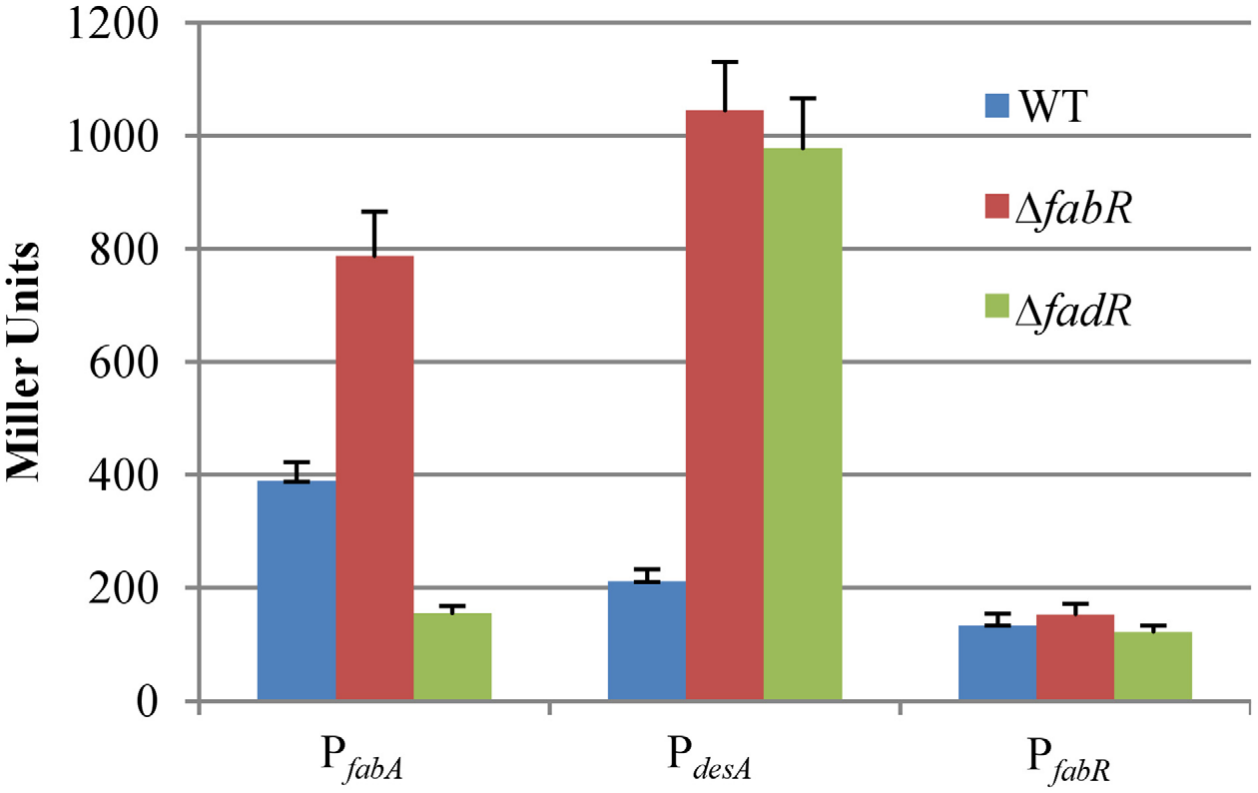
**Effects of FabR*_So_* and FadR*_So_* on expression of *fabA_So_, desA_So_*, and *fabR_So_***. The *fabA_So_* and *desA_So_ lacZ* reporters were described in Figure 3 and the *fabR_So_ lacZ* reporter was similarly constructed. Experiments were conducted independently at least three times and standard deviations were presented as error bars.

In *E. coli*, both FadR_Ec_ and FabR_Ec_ directly regulate the *fabA_Ec_* gene whereas *P. aeruginosa* DesT_Pa_ represses expression of *desB* (Zhang et al., 2002, 2007; Zhu et al., 2009). To assess effects of FabR_So_ and FadR_So_ on expression of the *fabA_So_* and *desA_So_* genes *in vivo*, we measured the activities of the *fabA_So_* and *desA_So_* promoters using the *lacZ*-reporters described above (Figure 4). In the absence of FabR_So_, β-galactosidase activities driven by the *fabA_So_* and *desA_So_* promoters increased ~2- and 5-fold, respectively, suggesting that the regulator functions as a repressor for both genes. FadR_So_, however, displayed an opposite effect on expression of the *fabA_So_* and *desA_So_* genes, at respective ~40% and ~5-fold relative to the wild type levels in its absence. This observation suggests that FadR_So_ acts as an activator for the anaerobic pathway and a repressor for the aerobic pathway. Additionally, we examined whether FabR_So_ autoregulates its own expression given that its coding gene shares the intergenic region with the *desA_So_* gene. A fragment of ~300 bp upstream of the *fabR_So_* gene, covering the intergenic region separating these two genes (144 bp), was amplified and placed in front of the *E. coli lacZ* gene to construct the P*_fabR_*-lacZ reporter. By using this system, we found that the *fabR_So_* promoter activity seemed constitutive, hardly affected by loss of either FabR_So_ or FadR_So_ (Figure 4).

### FadR_SO_ directly controls *fabA_SO_* but not *DesA_SO_*

**Figure 5:**
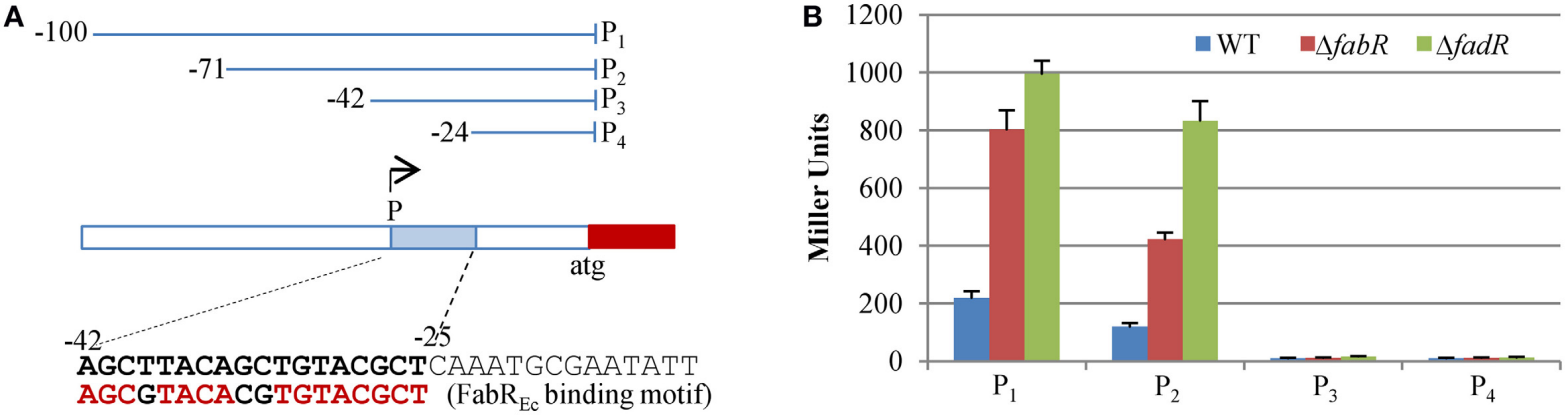
**The *desA_So_* promoter is immediately before a possible FabR*_So_*-binding motif. (A)** Constructs used to determine the *desA_So_* promoter location. The sequence (-AGCTTACAGCTGTACGCT-) resembles the *E. coli* FabR*_So_*-binding motif (presented underneath for comparison). **(B)** Activities of four constructs in **(A)** in WT, Δ*desA_So_* and Δ*fabA_So_* strains. Experiments were conducted independently at least three times and standard deviations were presented as error bars.

As shown above, loss of FadR_So_ or FabR*_So_* results in significantly altered expression of the *fabA_So_* and *desA_So_* genes. According to the prediction made by NNPP, the most confident *desA_So_* promoter starts at the “A” of −42 relative to the translation initiation code ATG. To confirm this, a series of fragments varying in length were amplified and placed before the *E. coli lacZ* gene for the promoter activity assay (Figure 5A). In the background of the wild-type, the fragments of P_1_ (up to −100 bp) and P_2_ (up to −71 bp) were able to drive the β-galactosidase production at levels comparable to that with the fragment of ~300 bp as shown in Figures 3, 4, 5B, indicating that the promoter is included in both P_1_ and P_2_. In contrast, the activities of the β-galactosidase were not detected with P_3_ (up to −42 bp) or P_4_ (up to −24 bp), indicating that these fragments do not cover the intact promoter. Moreover, all fragments tested behaved as expected in the *fadR_So_* and *fabR_So_* deletion strains (Figures 4, 5B). These data support that the promoter lies between −71 and −42, in perfect agreement with the prediction. It is worth noting that the sequence of 18 bp starting with the predicted transcriptional starting nucleotide “A” closely resembles the FabR_Ec_-binding motif (-AGCGTACACGTGTTCGCT-, the same nucleotides are underlined) (Feng and Cronan, 2011), implying that the *desA_So_* gene is under direct repression of FabR_So_.

**Figure 6:**
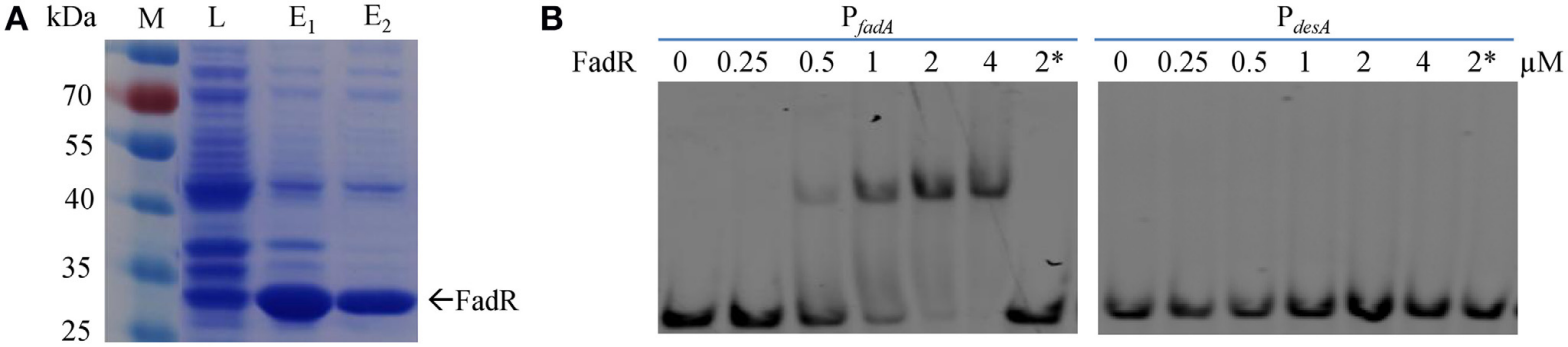
**FadR_So_-binding to selected promoters by EMSA. (A)** Overproduced and purified recombinant *S. oneidensis* His_6_-FadR_So_ from *E. coli* BL21 cells. M, protein marker; L, lysates; E_1_, elute from the first round purification; E_2_, elute from the second round purification; **(B)** Interaction of P*_fadA_* and P*_desA_* DNAs with His_6_-FadR_So_. The probes were prepared by PCR with end-labeled primers. EMSA was performed with 2 nM end-labeled probes and various amounts of proteins. Non-specific competitor DNA (2 μg/μl poly dI·dC) was added to the lane with 2 μM protein and specific competitor (100 nM unlabeled P_fadA_ and P_desA_ probes) was added to the lane with 2 μM protein marked by an asterisk.

Although, expression of the *desA_So_* gene is substantially upregulated in the absence of FadR_So_ (Figure 4), its regulation by FadR may be indirect as the increased *desA_So_* expression may be a result of the reduced production of FabA, a phenomenon shown in Figure 3. To test whether FadR_So_ and/or FabR_So_ interacts with DNA fragments upstream of the *fabA_So_* and *desA_So_* genes, we employed a DNA-binding gel shift assay. Both FadR_So_ and FabR_So_ with the N-terminal His-tag were subjected to expression and purification from *E. coli*. Soluble FadR_So_ was obtained smoothly (Figure 6A). In contrast, we were unable to purify FabR_So_ after many attempts, a scenario reported before with *E. coli* and *Vibrio cholerae* FabR, implicating that the same frustrating properties are shared by these proteins (Feng and Cronan, 2011). The DNA fragments, ~300 bp in length centered by the predicted promoter of the genes to be tested, were prepared by PCR. It was found that FadR_So_ significantly reduced the motility of the fragments for *fabA_So_* at a protein concentration of 0.5 μM. The strongest binding was observed with the protein at 2 μM. The specific binding was validated by that the binding was completely blocked by excessive (50x) unlabeled the same probe but not blocked by non-specific competitor of 2 μg/μl poly dI·dC. In contrast, no interaction was found with the *desA_So_* promoter DNA (Figure 6B). These results manifest that FadR_So_ directly controls *fabA_So_* but not *desA_So_* although expression of both genes is significantly altered in its absence.

### FabR directly represses both *fabA* and *desA*

Given our inability to obtain soluble FabR_So_, we turned to Bacterial one-hybrid (B1H) system to investigate DNA-protein interaction *in vivo* in *E. coli* cells (Guo et al., 2009). Positive interaction between “bait” (DNA) and “target” (DNA-binding regulator) allows the reporter strain to grow on 3-amino-1,2,4-triazole (3-AT). To prepare the bait, a ~ 300 bp fragment of *fabA_So_* or *desA_So_* centered by the predicted binding sequence or the promoter sequence was cloned into pBXcmT, which was paired with pTRG carrying either the *fabR_So_* gene or the *fadR_So_* gene for co-transformation. The system was validated with positive control pairs (P_katB_/OxyR of *S. oneidensis*) and negative control plasmid pair (P_16S_/OxyR of *S. oneidensis*), which showed strong and no interaction in our previous work, respectively (Jiang et al., 2014; Li et al., 2014) (Table 3). While positive interactions from P_fabASo_/FadR_So_ were detected and confirmed by growth on plates containing both 3-AT and streptomycin (12.5 mg/ml), no colonies were obtained from P_desASo_/FadR_So_, supporting the EMSA result. With FabR_So_ as the target, both P*_fabASo_* and P_desASo_ generated positive results, suggesting that FabR_So_ likely interact with the intergenic sequence of both the *fabA_So_* and *desA_So_* genes.

**Table 3:** **Bacterial one-hybrid (B1H) assay of FabR_So_ and FadR_So_ with various *S. oneidensis* promoters**.

**Bait vector pBXcmT**	**Target vector pTRG**	**Colonies on NS plates^a^**	**Colonies on S plates^b^**	**C plates^c^**	**Interaction**
/–	/–	207	0	–	No
/–	/OxyR_So_	183	0	–	No
/P*_katB_*	/OxyR_So_	179	165	164	Yes (Positive control)
/P*_katB_*	/–	213	0	–	No
/P_16*S*_	/OxyR_So_	162	0	–	No (negative control)
/P_16*S*_	/FabR_So_	203	2	0	No (negative control)
/P*_fabA_*	/FabR_So_	158	154	153	Yes
/P*_desA_*	/FabR_So_	212	201	199	Yes
/P_16*S*_	/FadR_So_	177	0	0	No (negative control)
/P*_fabA_*	/FadR_So_	173	182	182	Yes
/P*_desA_*	/FadR_So_	193	1	0	Yes

a *NS plates, non-selective plates: M9 agar + 25 μg/ml chloramphenicol + 12.5 μg/ml tetracycline*.

b *S plates, selective plates: a + 5 mM 3-AT*.

c *C plates, confirmation plates: b + 12.5 μg/ml streptomycin*.

## Discussion

Bacteria often reside in the environment with constantly changing parameters, such as temperature, organic-solvent concentration, and pH, that require the immediate modification of existing membrane phospholipid acyl chains to optimize fitness under the new conditions. To control the production of a variety of fatty acids with different melting temperatures to achieve the proper physical state of the membrane phospholipids, the SFA, UFA, and BCFA biosynthesis pathways must be strictly regulated according to the availability of fatty acids (Zhang and Rock, 2008). In the present study, we show that facultative Gram-negative γ-proteobacterium *S. oneidensis* possesses both aerobic and anaerobic UFA synthesis pathways, a scenario reported before only in *P. aeruginosa* (Zhu et al., 2006). Both bacteria own a FabA-based anaerobic UFA synthesis pathway, which resembles the well-studied one from *E. coli*. In the case of the aerobic UFA synthesis pathway, however, significant differences are found. Unlike *P. aeruginosa*, which is equipped with two desaturases DesA and DesB, *S. oneidensis* contains only one. Based on the sequence similarity, the *S. oneidensis* desaturase is likely a counterpart of *P. aeruginosa* DesA. Moreover, DesA proteins in both bacteria appear to be sufficient for the desaturation reaction whereas *P. aeruginosa* DesB is predicted to be functionally associated with DesC, whose coding gene forms an operon with *desB*. However, with respect to synteny, the *S. oneidensis desA* is more closely related to the *P. aeruginosa desB*, both of which form a divergent with their repressive regulators, FabR and DesT, respectively (Zhu et al., 2006; Zhang et al., 2007). Given that FadR has not been identified in *P. aeruginosa, S. oneidensis* is a good research model for studying regulation of UFA biosynthesis in bacteria owning both aerobic and anaerobic UFA synthesis pathways.

In line with *P. aeruginosa*, the anaerobic pathway undoubtedly plays a dominant role in UFA biosynthesis as the loss of DesA barely affects the fatty acid composition whereas the profound changes result from the removal of FabA in *S. oneidensis* (Zhu et al., 2006). The major UFA found in *S. oneidensis* membrane is C16:1, consistent with previous reports (Venkateswaran et al., 1999; Abboud et al., 2005). Although bacteria commonly produce more UFAs as a means to increase membrane fluidity, it is not adopted by *S. oneidensis* as a major strategy. Instead, the enhanced fluidity is achieved by elevating the proportion of BCFAs (mainly iso- and antiiso-C15:0), from ~25% at 22 °C to ~74% at 3°C (Abboud et al., 2005). In the present study, we found that production of BCFAs is increased in reverse proportional to production of UFAs, supporting the predominant role of BCFAs in determining membrane fluidity. Consistent with the dominant role of the anaerobic pathway in UFA biosynthesis, this phenomenon was observed only when anaerobic rather than aerobic pathway is damaged, as in strains lacking either FabA or FadR.

Despite subordinate, the aerobic pathway is important in the absence of the anaerobic pathway. In addition to their role in maintaining membrane structure and function, UFAs are involved in many biological processes, some of which may yet be properly appreciated. For example, for the first time we showed here that cytochrome *c* biosynthesis is dependent on UFAs. As *S. oneidensis* produces more than 40 such proteins, which are largely responsible for its respiratory versatility (Meyer et al., 2004; Gao et al., 2010b; Jin et al., 2013; Fu et al., 2014b), such a finding implies that UFAs are critical for features that are currently exploited for application.

To be more effective in complementing to the anaerobic pathway, expression of the *desA* gene is substantially increased when FabA is depleted. Although we do not yet know how this is achieved, the underlying mechanism may also account for enhanced expression of *desA* in the absence of FadR. Our data obtained from both *in vivo* and *in vitro* analyses demonstrated that FadR directly activates transcription of the *fabA* gene but affects expression of *desA* in an indirect manner. Thus, increased expression of *desA* in the strain lacking FadR is probably a result of decreased production of FabA. FabR acts as a repressor for the *desA* gene, similar to DesT for *desB* in *P. aeruginosa* (Zhu et al., 2006; Zhang et al., 2007; Miller et al., 2010). Given that both DesT_Pa_ and FabR_So_ are homologs of *E. coli* FabR_Ec_, it is possible that these two proteins are functional counterparts. Both FabR_Ec_ and DesT_Pa_ were proposed to respond to the composition of fatty acids (the UFA:SFA ratio of the acyl-ACP pool) available for membrane phospholipid synthesis to adjust expression of relevant genes for properly balanced production of UFA and SFA (Zhu et al., 2009; Miller et al., 2010). However, this notion was challenged later, at least in the case of FabR_Ec_, by the finding that FabR_Ec_ primarily functions to report the presence of exogenous UFA (as their CoA esters) rather than the composition (Feng and Cronan, 2011). Our data manifested that no matter what FabR_So_ and DesT_Pa_ respond to, their regulatory effect is modest.

Unlike *E. coli*, which has the anaerobic pathway only, *S. oneidensis* and *P. aeruginosa* possess two pathways and the dominant role of the anaerobic pathway in UFA synthesis overwhelms the contribution of the aerobic pathway (Campbell and Cronan, 2001; Zhu et al., 2006). The minor effect of losing DesT_Pa_ can be readily explained because this regulator does not influence expression of the *fabA_Pa_* gene of the anaerobic pathway (Zhu et al., 2006). However, this is not the case in *S. oneidensis*. FabR_So_ acts as a repressor for both the *fabA_So_* and *desA_So_* genes. Although the expression of *fabA_So_* is elevated by ~2-fold in the Δ*fabR_So_* strain, the fatty acid composition is not noticeably altered, indicating that FabA produced at the wild-type level is sufficient to maintain balanced membrane lipid homeostasis. In the absence of FadR_So_, in contrast, the expression of *fabA_So_* is too low to ensure UFA production. Combining the finding that the strain lacking FadR_So_ overall suffers more severe impairment than that missing FabA_So_, we speculate that the regulatory scope of FadR_So_ is not limited to fatty acid biosynthesis. Efforts to define the regulon of FadR_So_ are underway.

### Conflict of interest statement

The authors declare that the research was conducted in the absence of any commercial or financial relationships that could be construed as a potential conflict of interest.
