# Supplementary material for: Transcription factors FabR and FadR regulate both aerobic and anaerobic pathways for unsaturated fatty acid biosynthesis in Shewanella oneidensis
Source: Front Microbiol. 2014 Dec 22;5:736. doi: 10.3389/fmicb.2014.00736 (PMC4273635; doi:10.3389/fmicb.2014.00736)
Supplement: Supplementary file 1 [file Presentation1.PDF]

Supplemental Materials of

Transcription factors FabR and FadR regulate both aerobic and anaerobic pathways for unsaturated fatty acid biosynthesis in *Shewanella oneidensis*

Qixia Luo, Miaomiao Shi, Yedan Ren, and Haichun Gao*

Institute of Microbiology and College of Life Sciences, Zhejiang University, Hangzhou, Zhejiang, 310058, China

Table S1. Primers used in this study

Primers	Primer sequences
**In-frame deletion**	
HG0197-5I	CCGCGATCCTCATATTATTGAGCCAGATGATAGGGGGTTT
HG0197-3I	CAATAATATGAGGATCGCGGGGAAGAGCCTGACAGCAACT
HG0198-5I	ATATCTATCTGGAGGGACGTGCACTAAGCTGATTAAAAGCCG
HG0198-3I	ACGTCCCTCCAGATAGATATAAGTGCTCGCCGATCAATTA
HG1856-5I	ATTCCTATGCCGATGGCGGTTGGCCACAGGCGATAAGTTC
HG1856-3I	ACCGCCATCGGCATAGGAATGTCGCCAAATTTACAGTGCAA
HG2885-5I	AGTGCCATCGTATTCCACGTGCAAGGTAGTCCGAGTCAC
HG2885-3I	ACGTGGAATACGATGGCACTCTATACAGTCGAGTCGGCC
HG0197-1856-5I	ATTCCTATGCCGATGGCGGTTGGCCACAGGCGATAAGTTC
HG0197-1856-3I	ACCGCCATCGGCATAGGAATGTCGCCAAATTTACAGTGCAA
**Complementation**	
HG0197-CF	GGAATTCGCCCCTTACGCATCATTTGC
HG0197-CF	CGGGATCCGCGGCCGTATCATTTTTACCG
HG1856-CF	GGGAATTCTCGTTAGCCATTACGGGAGC
HG1856-CF	CGGGATCCTTCCAAGCCGGTATTGGTGG
HG2885-CF	GGGAATTCCGCCTGACTCATTGTCATCG
HG2885-CR	CGGGATCCCTAGGGATAAGCAGAGCCGC
desA$_{Pa}$-CF	GGGGTACCGATGGCGATTGCGAGATAAGC
desA$_{Pa}$-CR	CCGCTCGAGACGCCGAAACGCAACG
***lacZ* reporters**	
P*fabA*-F	GGGAATTCAGAACAACCGATTATTCAGTCGTT
P*fabA*-R	CGGGATCCGTAATCATGGTCATTGTTTTACTACTCCAGCAATAAGA
P*desA*-P1-F	GGGAATTCTTTACGTTTGAAACACAGCCCCAT
P*desA*-P2-F	GGGAATTCTTGACCAAATAAGATTGCTAGGCTA
P*desA*-P3-F	AATTCAGCTTACAGCTGTACGCTCAAATGCGAATATTGGAAATTATAAT GACCATGATTACG
P*desA*-P4-F	AATTCAGCTTACAGCTGTACGCTCAAATGCGAATATTGGAAATTATAAT GACCATGATTACG
P*desA*-P-R	CGGGATCCGTAATCATGGTCATTATAATTTCCAATATTCGCATTTGAGC
**Protein expression**	
pET28a-*fadR*-F	CCGCCATGGGCCATCATCATCATCATCACGGCGGCATTATCAATGCCAA AGGACCTG
pET28a-*fadR*-R	CCGCTCGAGCTAATGGGAGTCCTGCTGTG

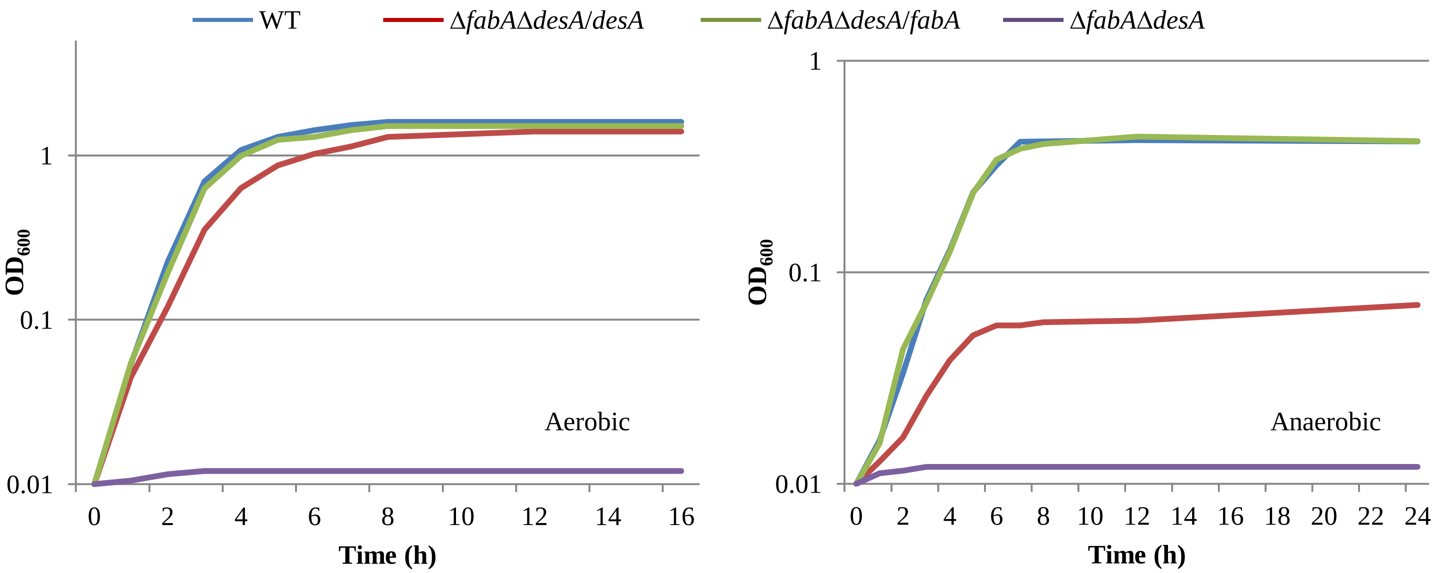

FIGURE S1 Genetic complementation in liquid media. Growth of WT and Δ*fabA*$_{So}$Δ*desA*$_{So}$ strains in liquid media under aerobic and anaerobic conditions. The double mutant was complemented *fabA*$_{So}$ or *desA*$_{So}$ *in trans*, with empty vector as control. Experiments were conducted independently at least three times and standard deviations (less than 5% of the means) were omitted for clarity.
